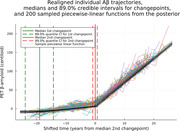# Bayesian changepoint models for amyloid‐centric synchronisation of biomarker trajectories in AIBL

**DOI:** 10.1002/alz.090615

**Published:** 2025-01-09

**Authors:** Martin Saint‐Jalmes, Daniel Beck, Pierrick Bourgeat, Colin L Masters, Benjamin Goudey

**Affiliations:** ^1^ ARC Training Centre in Cognitive Computing for Medical Technologies, Parkville, VIC Australia; ^2^ University of Melbourne, Parkville, VIC Australia; ^3^ Florey Institute of Neuroscience and Mental Health, University of Melbourne, Parkville, VIC Australia; ^4^ RMIT University, Melbourne, VIC Australia; ^5^ CSIRO, Brisbane, QLD Australia

## Abstract

**Background:**

The modelling of biomarker dynamics in Alzheimer’s Disease from cohort studies faces challenges due to the lack of clear temporal points of references and the natural variability across individuals. Mixed‐effects models are often used to account for individual differences, but a disease timescale can enable better population‐level modelling than age or time since enrolment. Previous literature explored the temporal synchronisation of patients through observed time of conversion to MCI or AD, amyloid positivity, or aligning cognitive trajectories. We propose an amyloid‐centric synchronisation approach that estimates patient‐specific time‐shifts to best align them on a population‐level piecewise linear trajectory.

**Method:**

We rely on 521 patients of the AIBL study with at least 3 measurements of PET amyloid, and model a 3‐segment, piecewise linear trajectory of centiloid over time. Our Bayesian model assumes that observed amyloid values are noisy realisations of the piecewise linear trajectory, and that the time of measurements is shifted by some individual‐specific lag. The first segment is constrained to have a zero slope, and individuals who were not observed to reach the first changepoint were excluded from subsequent analysis, as they may never develop Alzheimer’s Disease.

**Result:**

We identified a changepoint in the amyloid trajectory at a median of (89% credible interval: [14.45, 23.64]) after initial accumulation in shifted time. Patients with measurements falling within this credible interval have a median of 18.9 centiloid (range of 2.0 to 30.1). On average, this changepoint describes an acceleration in amyloid accumulation, switching from a median 0.75 centiloid/year [0.65, 0.87] rate to 6.38 centiloid/year [6.20, 6.56] (Figure 1).

**Conclusion:**

Synchronisation approaches allow for better group‐level assessments (such as the relative timing of biomarkers) rather than attributing variability to patient‐specific effects. Our approach was able to realign individuals on a common trajectory and amyloid‐centric timescale. Markov chain Monte Carlo sampling allows us to estimate uncertainties for all parameters: slopes and intercepts, changepoints, time‐shifts and the fit residual. Further work could explore the timing of other biomarker changes in this amyloid‐synchronised time.